# PP46 Budget Impact Analysis Of Tafenoquine Following The G6PD Test For Treatment Of Plasmodium Vivax Malaria In The Brazilian Amazon

**DOI:** 10.1017/S0266462325102651

**Published:** 2025-12-29

**Authors:** Marcia Pinto, Ivan Zimmermann, Márcia Gisele Costa, Ana Carolina Carioca da Costa

## Abstract

**Introduction:**

In Brazil, approximately 99 percent of malaria cases are concentrated in the Amazon region, 68 percent of which are due to *Plasmodium vivax.* Tafenoquine, a single-dose drug, should be offered with a glucose-6-phosphate dehydrogenase (G6PD) test because it can cause severe hemolysis in patients with G6PD deficiency. New technologies represent a promising strategy to reduce the burden of malaria in Brazil.

**Methods:**

From the perspective of the Unified Health System (SUS), we estimated the incremental budget impact of introducing tafenoquine and the G6PD test for diagnosing and treating *Plasmodium vivax* malaria in the Brazilian Amazon region over a five-year time horizon. A budget impact model was developed and two scenarios were analyzed: a reference scenario based on current Brazilian national antimalaria guidelines, and an alternative scenario incorporating the novel tafenoquine regimen and G6PD deficiency diagnosis. Face validity was assessed through consensus meetings with policymakers to ensure the model’s accuracy and transparency.

**Results:**

The incremental budget impact of adopting the G6PD test and the novel tafenoquine regimen was estimated at USD6.26 million over five years, assuming full implementation in the Brazilian Amazon region. The model predicted that both technologies would reduce hospitalization costs in approximately 22 percent of cases. The parameter with the greatest effect on the incremental budget impact was the price of the test kit. Additionally, material losses during diagnosis also significantly influenced the results.

**Conclusions:**

The G6PD testing and tafenoquine regimen are valuable strategies for reducing the malaria burden in the Brazilian Amazon Region and could generate potential savings for the SUS. Face validity was a key advantage for ensuring transparency throughout the budget impact development process and for providing an economic evaluation framework to optimize the use of health resources, thereby supporting healthcare decision-making.

